# Supplementary material for: Physical Activity Trajectories among Persons of Turkish Descent Living in Germany—A Cohort Study
Source: Int J Environ Res Public Health. 2020 Aug 31;17(17):6349. doi: 10.3390/ijerph17176349 (PMC7504423; doi:10.3390/ijerph17176349)
Supplement: Supplementary file 1 [file ijerph-17-06349-s001.pdf]

Supplementary Table 1. Weekly activity levels of PA trajectories and of the total sample (n=197, complete PA data for baseline and follow-up)

Weekly activity level	Total N=197		Inactive n=52 (23.7%)		Decreasing N=24 (12.2%)		Increasing N=83 (42.1%)		Stable active n=38 (19.3%)	
	baseline	follow-up	baseline	follow-up	baseline	follow-up	baseline	follow-up	baseline	follow-up
Inactive (no activity at all)	57.4	34.0	100	100	0	62.5	73.5	0	0	0
Light (<150min/week)	30.5	41.1	0	0	50.0	37.5	19.3	48.2	84.2	84.2
Moderate (150- <300min/week)	7.6	12.2	0	0	29.2	0	7.2	26.5	5.3	5.3
High (≥300min/week)	4.6	12.7	0	0	20.8	0	0	25.3	10.5	10.5
